# Progress and Gaps in Respiratory Disease Research and Treatment: Highlights of the IRM 2024 in Shanghai

**DOI:** 10.70322/jrbtm.2024.10021

**Published:** 2024-12-04

**Authors:** Jin-San Zhang, Qhaweni Dhlamini, Qiang Guo, Meiyu Quan, Jin Wu, Handeng Lyu, Lei Chong, Yuqing Lv, Yuting Lin, Bin Zhou, Yuru Liu, Honglong Ji, Xinhua Lin, Wen Ning, Pengfei Sui, Huaiyong Chen, Peisong Gao, Wei Chen, Xiaobo Zhou, Yuanlin Song, Chaoqun Wang, Xiao Su, Jinfu Xu, Jie Sun, Yin Chen, Yan Geng, Hai Song, Hongbin Ji, Yuanpu Peter Di, Hao Tang, Chao Lu, Jinghong Li, Ke Cheng, Mengshu Cao, Jiurong Liang, Yingze Zhang, Yang Zhou, Ying Xi, Weining Xiong, Bin Cao, Jianwen Que, Dianhua Jiang

**Affiliations:** 1 Zhejiang Key Laboratory of Interventional Pulmonology, and Medical Research Center, The First Affiliated Hospital of Wenzhou Medical University, Wenzhou 325035, China; 2 The State Key Laboratory of Cell Biology, CAS Center for Excellence on Molecular Cell Science, Shanghai Institute of Biochemistry and Cell Biology, University of Chinese Academy of Sciences, Chinese Academy of Sciences, Shanghai 200031, China; 3 Department of Pharmacology and Regenerative Medicine, University of Illinois College of Medicine, University of Illinois at Chicago, Chicago, IL 60607, USA; 4 Department of Surgery, Stritch School of Medicine, Loyola University Chicago, Maywood, IL 60153, USA; 5 State Key Laboratory of Genetic Engineering, School of Life Sciences, Greater Bay Area Institute of Precision Medicine (Guangzhou), Zhongshan Hospital, Fudan University, Shanghai 200032, China; 6 State Key Laboratory of Medicinal Chemical Biology, College of Life Sciences, Nankai University, Tianjin 300071, China; 7 Tianjin Key Laboratory of Lung Regenerative Medicine, Haihe Hospital, Tianjin University, Tianjin 300350, China; 8 Division of Allergy and Clinical Immunology, Johns Hopkins University School of Medicine, Baltimore, MD 21205, USA; 9 Division of Pulmonary Medicine, Children’s Hospital of Pittsburgh of UPMC, Department of Pediatrics, University of Pittsburgh School of Medicine, Pittsburgh, PA 15224, USA; 10 Brigham and Women’s Hospital and Harvard Medical School, Boston, MA 02115, USA; 11 Department of Pulmonary Medicine, Zhongshan Hospital, Fudan University and Shanghai Respiratory Research Institute, Shanghai 200032, China; 12 State Key Laboratory of Drug Research, Shanghai Institute of Materia Medica, Chinese Academy of Sciences, Shanghai 201203, China; 13 Unit of Respiratory Infection and Immunity, Shanghai Institute of Immunity and Infection, Chinese Academy of Sciences, Shanghai 200032, China; 14 Department of Respiratory and Critical Care Medicine, Shanghai Pulmonary Hospital, School of Medicine, Tongji University, Shanghai 200433, China; 15 Carter Immunology Center, University of Virginia, Charlottesville, VA 22908, USA; 16 School of Plant Science, University of Arizona, Tucson, AZ 85721, USA; 17 Key Laboratory of Carbohydrate Chemistry and Biotechnology, Ministry of Education, School of Life Sciences and Health Engineering, Jiangnan University, 1800 Lihu Avenue, Wuxi 214122, China; 18 Department of Respiratory and Critical Care Medicine, the Fourth Affiliated Hospital of School of Medicine, International School of Medicine, International Institutes of Medicine, Zhejiang University, Yiwu 322000, China; 19 Department of Environmental and Occupational Health, School of Public Health, University of Pittsburgh, Pittsburgh, PA 15261, USA; 20 Department of Respiratory and Critical Care Medicine, Changzheng Hospital, Naval Medical University, Shanghai 200003, China; 21 Department of Genetics and Development, Columbia University Irving Medical Center, New York, NY 10032, USA; 22 Department of Medicine, University of California San Diego, La Jolla, CA 92093, USA; 23 Department of Biomedical Engineering, Columbia University, New York, NY 10032, USA; 24 Department of Respiratory and Critical Care Medicine, Nanjing Drum Tower Hospital, Nanjing University Medical School, Nanjing 210008, China; 25 Department of Medicine, Cedars-Sinai Medical Center, Los Angeles, CA 90048, USA; 26 Division of Pulmonary, Allergy, and Critical Care Medicine, Department of Medicine, University of Pittsburgh, Pittsburgh, PA 15213, USA; 27 Department of Molecular Microbiology and Immunology, Brown University, Providence, RI 02912, USA; 28 School of Life Science and Technology, ShanghaiTech University, Shanghai 201210, China; 29 Department of Respiratory and Critical Care Medicine, Shanghai Ninth People’s Hospital, Shanghai Jiao Tong University School of Medicine, Shanghai 200011, China; 30 Department of Pulmonary and Critical Care Medicine, Center of Respiratory Medicine, China-Japan Friendship Hospital, Beijing 100029, China; 31 Columbia Center for Human Development, Columbia University Irving Medical Center, New York, NY 10032, USA; 32 Department of Biomedical Sciences, Cedars-Sinai Medical Center, Los Angeles, CA 90048, USA

**Keywords:** Lung disease, Epithelial repair, Lung development, Pulmonary fibrosis, Therapy

## Abstract

Respiratory diseases pose a major public health challenge globally, necessitating collaborative efforts between basic researchers and clinicians for effective solutions. China, which is heavily impacted by a broad spectrum of respiratory disorders, has made notable strides in both research and clinical management of these diseases. The International Respiratory Medicine (IRM) meeting was organized with the primary goal of facilitating the exchange of recent research developments and promoting collaboration between Chinese and American scientists in both basic and clinical research fields. This article summarizes key insights from IRM2024, held in Shanghai, where a wide range of topics were discussed, including lung tissue development, disease mechanisms, and innovative therapeutic strategies. By integrating perspectives from basic, translational, and clinical research, IRM2024 highlighted recent advancements, addressed persistent challenges, and explored future directions in respiratory science and clinical practice. The insights gained from IRM2024 are poised to be pivotal in shaping future research and therapeutic approaches, further reinforcing the global commitment to enhancing respiratory health and improving patient outcomes.

## 1. Introduction

The 2nd International Respiratory Medicine (IRM2024) meeting took place in Shanghai from 28–30 June 2024, showcasing the latest advancements and promising developments in respiratory research. This flagship event featured lectures by leading scientists affiliated with the Chinese-American Lung Association (CALA), an independent nonprofit organization committed to advancing research, patient care, education, and advocacy in pulmonary medicine. The conference was co-organized by CALA and the Editorial Board of *Chinese Medical Journal Pulmonary and Critical Care Medicine*, with Drs. Jianwen Que, Bin Cao, Weining Xiong, and Peifang Wei are the co-organizers, with Zhiyu Dai, Dianhua Jiang, Xiaobo Zhou, and Jie Sun serving as advisory committee members. Over the course of three days, more than 30 speakers delivered talks across six sessions, addressing a broad array of topics ranging from lung tissue development and disease mechanisms to therapeutic innovations. Each session concluded with interactive Q&A segments moderated by the session chairs, allowing for dynamic engagement between speakers and attendees. IRM2024 provided an invaluable platform for research exchange, fostering communication and networking opportunities for researchers at all career stages.

Despite extensive global research efforts at basic, translational, and clinical levels, respiratory diseases remain a major public health challenge. These conditions can be broadly categorized into communicable diseases, such as viral infections, and non-communicable diseases, including chronic obstructive pulmonary disease (COPD), asthma, lung cancer, and interstitial lung diseases. Chronic respiratory conditions, particularly COPD and asthma, continue to significantly contribute to the global burden of non-communicable diseases, with environmental, occupational, and behavioral exposures being the most common risk factors.

In recent years, China has made remarkable progress in respiratory research, driven by rapid technological advancements and increased funding from organizations like the National Natural Science Foundation of China (NSFC). A substantial portion of this research success can be attributed to international collaborations, highlighting the critical role of global partnerships in addressing the worldwide burden of chronic respiratory diseases. This summary highlights the diverse range of research presented by speakers across various fields of respiratory medicine at the IRM2024 conference.

## 2. Lung Repair and Regeneration

The conference began with a keynote address by Dr. Bin Zhou, who presented a comprehensive overview of his research on lineage-tracing studies, focusing on identifying and characterizing injury-induced lung stem and progenitor cells. His work also revealed mechanistic insights into the signaling pathways influencing stem cell behavior and regenerative processes. Zhou highlighted his recent research uncovering the origin and fate of injury-induced p63^+^ progenitors following lung injury. Using sequential genetic lineage tracing, Zhou and colleagues identified a CC10^+^ airway secretory cell-derived p63^+^ progenitor that plays a significant role in alveolar regeneration following bleomycin-induced lung injury in mice [1]. Clonal and single-cell trajectory analyses of p63 lineage-positive cells from bleomycin-injured mice demonstrated that these progenitors proliferate and differentiate into alveolar type 1 (AT1) and type 2 (AT2) cells through distinct pathways.

Zhou emphasized the importance of precisely identifying stem and progenitor populations responsible for lung maintenance and repair, which is crucial for developing reparative strategies for lung diseases. Several lineage-tracing studies have implicated multiple cell types, including airway-derived progenitors and AT1 cells, as sources of AT2 cells in various alveolar injuries [2,3]. However, the specificity of the lineage-tracing tools used in some studies has been questioned. For example, earlier studies using the Hopx-CreER line to target AT1 cells suggested that AT1 cells can regenerate into AT2 cells after alveolar injury. However, Zhou’s more recent work demonstrated that the Hopx-CreER tool also labels AT2 cells and other known AT2 progenitors, such as bronchioalveolar stem cells (BASCs) and club cells, underscoring the need for improved genetic lineage-tracing tools with enhanced precision [4]. Additionally, using dual-recombinase intersectional strategies, this study clarified that AT1 cells do not give rise to AT2 cells during homeostasis or after injury.

AT2 cells function as progenitor cells and can self-renew and give rise to AT1 to maintain alveolar epithelial homeostasis and repair following injury [5,6]. The importance of AT2 cells in lung repair and disease was emphasized in several talks during the conference. Dr. Yuru Liu presented an insightful study on a novel subpopulation of CD44^+^ AT2 cells and its role in lung homeostasis and repair [7,8]. This subpopulation, which constitutes about 3% of AT2 cells in mice, exhibits distinct characteristics, including higher proliferation rates and enhanced differentiation capabilities, making it effective in forming alveolar organoids. Notably, CD44^+^ AT2 cell numbers increase with age, although their progenitor properties decline. CD44^+^ AT2 cells possess a unique gene expression profile, including immune response markers, and are predominantly located near macro blood vessels, where they may be regulated by IL1β-NFkB and STAT1 signaling. LYVE1^+^ blood vessel endothelial cells and Gli1^+^ adventitial fibroblasts appear to support CD44^+^ AT2 cells by paracrine factors and contributing hyaluronic acid to maintain the niche microenvironment. Moreover, CD44^+^ AT2 cells are highly sensitive to KRAS transformation and respond robustly to Pseudomonas aeruginosa-induced injury. The study emphasizes the functional diversity of alveolar epithelial cells, including AT2 cells, and highlights the need for more refined models and techniques to explore lung repair mechanisms.

Dr. Jin-San Zhang presented recent advances in the characterization of a novel AT2 cell subpopulation in mice, termed injury-activated alveolar progenitors (IAAPs). This subpopulation is distinguished by low expression levels of *Sftpc, Sftpb*, *Fgfr2b and Etv5*, while being highly enriched for *Pd-l1* expression. Zhang’s team demonstrated that IAAPs represent quiescent, immature AT2 cells during homeostasis, which expand and differentiate into mature AT2 and AT1 cells after injury [9]. Moreover, a similar population of PD-L1^+^ AT2 cells exists in normal human lungs and is found aberrantly expanded in lungs affected by idiopathic pulmonary fibrosis (IPF) [10,11]. Furthermore, Fgf10/Fgfr2b signaling may play a crucial role in fibrosis resolution by promoting the proliferation of IAAPs and supporting IAAP-driven alveolar epithelial regeneration in mice [12]. Using the Cre/Dre dual-recombinase reporter system and Diphtheria Toxin A-mediated lineage ablation, the team provided evidence that IAAP mobilization following bleomycin-induced injury contributes to alveolar epithelial repair and regeneration. These findings underscore the therapeutic potential of harnessing IAAPs to treat lung diseases, such as IPF, offering new insights into regenerative medicine for pulmonary disorders.

Dr. Hong-Long Ji and colleagues provided a proteomics-based analysis of extracellular vesicles (EV) of bronchoalveolar lavage in acute lung injury patients, integrating human proteomics data to identify differentially expressed proteins and signatures corresponding to varying stages of acute respiratory distress syndrome (ARDS) [13]. These signatures, known as endotypes, were translated into clinical variables, enabling clinicians to predict the outcomes of ARDS patients based on routine data collected at enrollment. This EV proteomics-based approach offers a new avenue for clinical management of ARDS.

## 3. Lung Development and Homeostasis

Adult tissue regeneration often mirrors embryonic development, making a thorough understanding of the cellular and molecular mechanisms regulating lung development critical for therapeutic insights. Dr. Xinhua Lin discussed the pivotal role of the chromatin remodeler Znhit1 in lung development and homeostasis. Znhit1, which controls the deposition of the histone variant H2A.Z, is essential for lung branching morphogenesis [14]. Studies in Znhit1-deficient mice revealed that the absence of Znhit1 impairs lung branching due to the overactivation of the BMP signaling pathway, a key regulator of lung architecture during embryonic development. Rescue experiments using Noggin, a BMP antagonist, demonstrated that inhibiting BMP4 signaling could partially reverse the branching defects in Znhit1 mutant lung epithelial organoids. Znhit1 also plays a crucial role in postnatal lung homeostasis [15]. In AT2 cells, Znhit1 deficiency impairs alveolar regeneration and leads to fibrosis following lung injury, disrupting the transitional phase of AT2 differentiation and affecting cell proliferation. Additionally, Znhit1 deficiency alters chromatin organization and gene expression in alveolar macrophages, reducing their number and metabolic function, though macrophage differentiation remains unaffected. In summary, Znhit1 is essential for lung development and regeneration by regulating chromatin remodeling and BMP signaling, providing key insights into the mechanisms of lung disease and repair.

Dr. Wen Ning emphasized the critical role of follistatin-like 1 (Fstl1) in alveolar development, particularly alveologenesis. Fstl1 conditional knockout (CKO) mice exhibited severe alveolar simplification by day 7, underscoring the importance of Fstl1 in maintaining lung structure and function. Fstl1 interacts with the TGF-β1/BMP4 signaling pathway, regulating mesenchymal myofibroblasts (MyoFBs) and muscle tissue development during lung formation [16-18]. Disruptions in these pathways in Fstl1^−/−^ mice result in reduced airway smooth muscle (ASM) and αSMA levels, causing alveolar simplification and impaired lung function, similar to conditions like neonatal atelectasis. These findings position Fstl1 as a potential therapeutic target, particularly for diseases such as bronchopulmonary dysplasia (BPD) and COPD, by promoting healthy alveolar development and preventing structural lung degeneration.

Dr. Pengfei Sui explored the crucial role of metabolic regulation in determining lung stem cell fate, particularly in the context of COPD. He highlighted how alterations in cellular metabolism drive the differentiation of lung epithelial stem cells, contributing to disease progression. Specifically, the inhibition of pyruvate metabolism impairs basal cell differentiation, a key factor in COPD development. This discussion aligns with broader stem cell biology insights, suggesting that metabolism acts as a central regulator of stem cell fate decisions [19,20]. Previous studies have shown that metabolic pathways influence cell proliferation, quiescence, and stress responses, often through chromatin modifications and nutrient-sensitive signaling pathways like mTORC and AMPK. In lung diseases, metabolic imbalances may lead to stem cell exhaustion and impaired tissue regeneration. Pengfei also explored how targeting the YAP signaling pathway could reverse abnormal epithelial differentiation and restore lung function, presenting new therapeutic avenues. These findings underscore the growing recognition of the metabolic-epigenetic link in stem cell regulation and lung disease management.

Dr. Huaiyong Chen discussed the metabolic dynamics of lung stem/progenitor cells in lung diseases such as asthma and pulmonary fibrosis. He highlighted the interaction between club cells and eosinophils in asthma, where eosinophil-derived arachidonic acid metabolites inhibit club cell proliferation and promotes their differentiation into goblet cells, contributing to airway remodeling. Chen also discussed the critical role of glycolytic metabolism in the proliferation of both club cells and AT2 cells, aligning with studies that highlight the importance of glucose metabolism in maintaining progenitor cell pools during inflammation [21]. Autophagy regulates glycolysis and fatty acid synthesis pathways in AT2 cells, facilitating epithelial repair and inhibiting fibrosis. This finding aligns with studies on the role of autophagy in reprogramming metabolism to support tissue regeneration following lung injury [22]. Furthermore, metabolic disruptions, including impaired glutamine metabolism, reduce AT2 cell proliferation and differentiation, as seen in fibrosis models [23]. These insights suggest that targeting metabolic pathways, such as autophagy and glycolysis, may offer therapeutic strategies for lung diseases, including asthma and pulmonary fibrosis.

## 4. Biology of Asthma

The hallmark features of allergic asthma include airway hyperresponsiveness and inflammation [24]. Dr. Peisong Gao discussed the role of RhoA in Club cells and its contribution to airway inflammation. RhoA is a critical player in the induction of allergic inflammation and asthma pathogenesis [25]. Previous studies demonstrated that deleting RhoA in AT2 cells exacerbated airway hyperresponsiveness and inflammation through SLC26A4 in a murine asthma model [26]. However, more recent studies have shown that deleting RhoA in Clara cells has the opposite effect, reducing inflammation [27]. This protective mechanism involves stromal macrophages, a population of lung cells regulated by RhoA in CC10^+^ Clara cells. In patients with IPF, the expression of SPRR2A is upregulated in Clara cells, and the absence of SPRR2A has been linked to increased severity of allergic airway inflammation and epithelial cell dysfunction. Single-cell RNA sequencing (scRNA-seq) identified interstitial microphage-derivedCCL24 as the primary ligand altered in SPRR2A knockout mice after allergen exposure. Moreover, SPRR2A knockout mice exhibited distinct changes in bacterial abundances within bronchoalveolar lavage fluid (BALF) and the gastrointestinal tract. These findings underscore the significant role of the RhoA/Rho-kinase pathway in asthma pathophysiology and suggest that targeting RhoA/Rho-kinase signaling may offer a promising therapeutic strategy for asthma treatment.

Dr. Wei Chen discussed the application of multi-omics analysis and single-cell sequencing in lung diseases and immunological research, emphasizing how these technologies can identify disease-related genes and establish diagnostic and predictive models. A meta-analysis of genome-wide association studies (GWAS) involving over 4000 Latino children and adolescents with asthma identified a novel SNP in FLJ22447 (rs2253681), significantly associated with an increased risk of severe asthma exacerbations [28]. This SNP was also linked to DNA methylation of a cis-CpG site in FLJ22447 in the nasal epithelium, which in turn influences the expression of KCNJ2-AS, a gene implicated in atopic asthma. Genome-wide DNA methylation studies in nasal epithelium have revealed potential methylation markers of atopy and atopic asthma in children located near genes associated with immune regulation or airway epithelial integrity [29]. Additionally, scRNA-seq analysis has uncovered novel molecular signatures characterizing ARDS, a severe clinical condition associated with excessive inflammation [30]. A comparative scRNA-seq analysis between pneumonia patients with sepsis + ARDS and those with sepsis only but at risk of developing ARDS revealed distinct molecular signatures in their peripheral blood immune cells, offering potential biomarkers to differentiate ARDS from other respiratory complications. Using spatial transcriptomic integration analysis with scRNA-seq and scATAC-seq, dynamic changes in helper T cells and their corresponding chemokines were observed in the lungs following bacterial infection in mice [31].

Dr. Xiaobo Zhou explored the genetic basis of asthma, focusing on the 17q21 region due to its strong association with viral infections and asthma [32]. The interferon (IFN) response pathway, induced by RNA and DNA viruses, is a critical area of study in this region. Specifically, the Gasdermin B (GSDMB) gene, closely associated with interferon signaling, enhances the IFN response by activating TBK1 and interacting with the MAVS protein. Overexpression of GSDMB promotes IFN responses and exacerbates inflammation following RNA virus infections in mice and upon increased mitochondrial DNA release in asthmatic airway epithelial cells [33]. These findings provide new insights into asthma pathophysiology and may guide future drug development targeting asthma genes involved in interferon pathway regulation. Ongoing research also examines how genetic variations influence individual responses to environmental factors, which can affect asthma incidence and severity. Further investigation in this area could uncover new therapeutic targets for asthma.

## 5. Other Airway Diseases

Type 2 inflammation plays a key role in a broad range of diseases, including asthma and COPD. Dr. Yuanlin Song reported that IL-4, IL-13, and IL-5 are pivotal in asthma pathogenesis. These cytokines, key mediators of Type 2 inflammation, exhibit both distinct and overlapping roles [34]. IL-4 and IL-13 are essential for B-cell class switching, IgE synthesis, and T-cell polarization, while also impairing airway epithelial barrier function [35]. Additionally, these cytokines assist in eosinophil migration to tissues, with increased MUC5AC expression exacerbating mucus obstruction in asthmatics [36]. IL-13, in particular, plays a significant role in airway remodeling, contributing to smooth muscle growth, contractility, and epithelial fibrosis. Approximately 40% of COPD patients also exhibit Type 2 immune responses, highlighting the importance of these cytokines in both asthma and COPD.

Dr. Chaoqun Wang discussed the role of the Sonic Hedgehog (Shh) antagonist, Hedgehog-interacting protein (Hhip), in alveolar development and its therapeutic potential for BPD. Differential activation of Shh signaling was identified as a key molecular mechanism in lung development and homeostasis. Abnormal Shh signaling in the distal lung promotes the expansion of tissue-resident lymphocytes by inducing IL-7 production, which inhibits alveolar stem cell growth via IFNγ, contributing to emphysema development. Hhip plays a critical role in this process, with mesenchymal-specific deficiency of Hhip leading to emphysema [37]. In BPD mouse models, Hhip expression was significantly reduced, but administering Hhip-Fc protein improved alveolar development, indicating its potential as a therapeutic target for BPD.

Dr. Xiao Su explored the intricate interplay between the nervous and immune systems in the respiratory tract, with a focus on neuroimmune recognition mechanisms that help the body respond to respiratory threats such as pathogens and pollutants [38]. The activation of the α7 nicotinic acetylcholine receptor (α7nAChR) regulates inflammatory responses and viral infections [39]. For example, vagotomy and deletion of the Chrna7 gene significantly reduced lung damage in influenza virus infections. Conversely, α7nAChR activation promotes autophagy and ferroptosis, limiting Zika virus replication. Furthermore, α7nAChR signaling enhances influenza infection severity via the PTP1B-NEDD4L-ASK1-p38MAPK pathway, offering new insights for developing targeted therapies for respiratory diseases [40].

Dr. Jinfu Xu provided novel insight into our understanding of allergic bronchopulmonary aspergillosis (ABPA), which was first characterized in 1952 as a hypersensitivity reaction to *Aspergillus fumigatus* sensitization [41]. The persistent presence of *A. fumigatus* in the airways, coupled with a heightened Type 2 immune response, are the two key pathogenic factors of ABPA. Patients with ABPA who exhibit excessive mucus production and eosinophils in their bronchoalveolar lavage fluid release extracellular traps, one of the main causes of mucus plugging in these patients. The CARD9S12N homozygous mutation enhances RelB activity and IL-5 production in ABPA patients, promoting the Type 2 immune response. Th2-associated T peripheral helper (Tph) cells in ABPA patients induce the production of large amounts of IgE, underscoring the immune dysregulation driving the disease [42]. Understanding these factors is crucial for developing targeted therapies for ABPA, particularly in addressing mucus obstruction and immune imbalance.

## 6. Microbiome and Lung Diseases

Addressing the immune-epithelial niche has significant implications for managing post-viral lung sequelae. Dr. Jie Sun discussed the immune mechanisms underlying post-COVID-19 lung sequelae, particularly pulmonary fibrosis. Sun’s research identified a persistent immune response involving CD8-positive T cells and KRT5/KRT8-positive transitional progenitor cells in areas of abnormal lung repair. These immune and epithelial cell interactions play a crucial role in maintaining chronic inflammation and fibrosis. Using a mouse model, Sun and colleagues demonstrated that removing CD8-positive T cells post-infection improved lung regeneration, while their dysfunction led to prolonged lung damage, including airway and alveolar metaplasia. Their findings suggest that dysregulated immune responses and improper epithelial repair post-infection contribute to tissue fibrosis [43]. CD8-positive cells stimulate macrophages to produce IL-1β, which perpetuates abnormal epithelial progenitor differentiation and hinders recovery. By targeting immune-epithelial interactions, specifically the CD8^+^ T cell-macrophage axis, there is potential for novel therapies to promote lung repair after viral infections such as COVID-19.

Exploratory research by Dr. Yin Chen revealed the significant role of fungal exposure, particularly *Alternaria* spores, in lung diseases. The study demonstrated that exposure to fungal allergens induces inflammatory responses and interferon-related gene expression in lung tissue. *Alternaria* spores produce double-stranded RNA-like structures that activate Toll-like receptor 3 (TLR3), triggering immune responses. Blocking the interferon pathway during infection escalated the inflammatory process, leading to goblet cell metaplasia and heightened airway hyperresponsiveness, characteristic of asthma pathology. Further studies showed that fungal spores and metabolic pathways can affect immune and lung epithelial cell functions, often enhancing pro-inflammatory cytokine production while impairing antiviral responses. These findings suggest that fungal exposure not only exacerbates asthma but may also contribute to other diseases, including tumorigenesis, by inducing DNA damage and mutations [44,45]. This highlights the importance of considering fungal exposure as a critical factor in both asthma research and broader lung disease management, particularly in the context of gene-environment interaction. Dr. Yan Geng discussed the relationship between the lung microbiome and the progression of IPF. Recent studies highlight the pivotal role that disruptions in the lung microbiota may play in disease progression. In particular, *Stenotrophomonas* were significantly enriched in the lung tissue of IPF patients, contributing to the exacerbation of fibrosis. These findings are consistent with broader research showing that lung dysbiosis is associated with increased alveolar inflammation and fibrosis. Previous studies have demonstrated that lung bacterial burden predicts disease progression in IPF and that specific microbiota are linked to profibrotic immune responses [46].In animal models, the absence of lung microbiota protects against fibrosis and modulates both humoral and cellular lung immunity, reinforcing the connection between dysbiosis and disease severity [47]. Together, these studies suggest that targeting the lung microbiota could offer novel therapeutic strategies for managing IPF.

## 7. Lung Cancer and Treatment

Therapeutic strategies targeting EGFR and Notch pathways to manage lung diseases and prevent tumorigenesis have broad implications for respiratory medicine. Dr. Hai Song discussed the role of pulmonary neuroendocrine cells (PNECs) in lung injury and tumorigenesis, focusing on their regulation by EGFR signaling. PNECs, which express markers such as CGRP and ASCL1, are primarily located at airway bifurcations and are linked to diseases like small cell lung cancer (SCLC) and COPD. Using a CGRP-CreER^/+^ mouse model, Song and colleagues demonstrated that PNECs exhibit plasticity, transforming into other lung cell types, such as club and ciliated cells, during repair processes following lung injury. This transformation is driven by the activation of key signaling pathways, notably EGFR and Notch. His research suggests that PNECs share a lineage with alveolar cells and play a significant role in both lung development and repair. Importantly, Notch signaling was highlighted as critical for PNEC trans-differentiation, influencing their capacity to replenish damaged tissues [48]. These findings underscore the dual role of EGFR signaling in promoting both lung repair and tumor growth in SCLC.

Dr. Hongbin Ji highlighted the critical role of adeno-to-squamous transition (AST) in driving resistance to KRAS inhibitors, particularly in lung adenocarcinoma patients with KRAS-G12C and LKB1 mutations [49]. Ji and colleagues discovered that high expression of squamous cell carcinoma (SCC) markers prior to treatment correlates with poor responses to the KRAS inhibitor adagrasib. They demonstrated that AST, driven by the ELF5-DNp63 axis, enables tumors to evade KRAS inhibition. Using genetically engineered mouse models (GEMMs) and patient-derived organoids, they showed that tumors with KRAS-G12C mutations and LKB1 deficiencies undergo AST, reducing their dependence on KRAS signaling. These tumors acquire a plastic state, marked by high KRT6A expression and an AST plasticity signature, which further predicts poor drug response. This research underscores the importance of identifying biomarkers like AST plasticity and KRT6A to predict responses to KRAS-targeted therapies. It also provides deeper insights into how epigenetic reprogramming, particularly via ΔNp63, promotes squamous transition and drug resistance. It opens avenues for combination therapies targeting AST pathways to overcome resistance in KRAS-G12C-mutant lung cancers.

Dr. Jianwen Que highlighted the novel role of pulmonary sympathetic nerves in SCLC progression. Sympathetic nerves accumulated in SCLC lesions. Chemical or genetical blockage of sympathetic nerve function can suppress tumor growth, reduce proliferation, and increase cell death in SCLC, a highly lethal cancer with a global toll of 250,000 new cases and 200,000 deaths annually. These findings align with neural activity studies in cancer, where neuronal signaling has been shown to drive tumor growth and metastasis [50,51]. For instance, optogenetic stimulation of neurons promotes SCLC brain metastasis growth, while sympathetic nerve activity supports tumor angiogenesis and immune cell recruitment, facilitating metastasis. Targeting neural signaling, such as 6-hydroxydopamine (6-OHDA) or PKA inhibitors, offers promising therapeutic avenues by disrupting neuro-immune interactions and tumor proliferation in SCLC and pre-metastatic lung environments.

Dr. Yuanpu Peter Di discussed the impact of lipopolysaccharide (LPS)-induced chronic inflammation on lung cancer, particularly in the context of cigarette smoke carcinogen nicotine-derived nitrosamine ketone (NNK)-induced tumorigenesis. His research showed a more than sixfold increase in lung tumors in mice subjected to combined LPS and NNK treatments, likely due to inflammation-driven T lymphocyte infiltration and upregulation of the PD-L1 pathway, which fosters an immunosuppressive tumor microenvironment. Other studies revealed that chronic lung inflammation, such as that seen in COPD, elevates cancer risk by promoting K-ras mutations and creating immunosuppressive conditions [52]. Persistent inflammation is linked to T-cell exhaustion and the accumulation of myeloid-derived suppressor cells (MDSCs), which contribute to tumor progression and immune evasion [53]. These findings highlight the critical role of inflammation in both the onset and progression of lung cancers, particularly among smokers.

Dr. Hao Tang presented recent clinical research on EGFR-TKI drugs, emphasizing how precision treatments for lung cancer have extended patients’ progression-free survival (PFS) and overall survival (OS). From the first to third generation, EGFR-TKI drugs have provided more treatment options for lung cancer patients. Befotinib, a novel third-generation EGFR-TKI, has shown considerable efficacy in both first-line and second-line treatments for EGFR-mutant non-small cell lung cancer (NSCLC), including systemic and intracranial benefits. The use of TKIs in postoperative adjuvant therapy is also a focus of clinical research, with drugs like Osimertinib and Icotinib currently having indications for this purpose. Clinical trials have shown that Icotinib has a lower incidence of ≥Grade 3 adverse reactions compared to Osimertinib. EGFR-TKI combination therapies continue to be explored, with the FLAURA2 study showing that Osimertinib combined with or without platinum-containing chemotherapy extended PFS in EGFR-mutant NSCLC patients compared to monotherapy. The A+T model has also shown PFS benefits for first-generation TKIs, though no overall survival benefit was observed.

Dr. Chao Lu explored the intricate relationship between metabolic reprogramming and chromatin vulnerabilities in lung adenocarcinoma (LUAD), with a particular focus on tumors exhibiting aberrant activation of the NRF2 antioxidant pathway. His research revealed that NRF2 activation sensitizes LUAD cells to the inhibition of class I histone deacetylases (HDACs). By inhibiting HDACs, significant changes in chromatin structure and metabolic gene expression were observed, leading to a reduction in the flux through critical metabolic pathways essential for the survival of NRF2-active cancer cells [54].

## 8. Advances in Lung Disease Diagnosis and Therapy

As the field of respiratory medicine continues to evolve, significant advancements are being made in both diagnosis and treatment. Dr. Jinghong Li discussed the complex condition of Long COVID, characterized by persistent symptoms such as fatigue, breathlessness, and cognitive impairment, which severely impact quality of life [55]. The underlying mechanisms include vascular damage, immune dysregulation, and potential viral persistence. Effective management of Long COVID requires a multidisciplinary approach, with pulmonary rehabilitation emerging as a key intervention, especially for patients with respiratory symptoms. Li also emphasized the transformative role of innovative technologies like telemedicine, wearable devices, and telerehabilitation. These advancements enable continuous patient monitoring and offer a scalable, patient-centric approach to recovery. As future directions focus on refining wearable technologies and enhancing predictive capabilities through machine learning, the potential for personalized, technology-driven care for Long COVID patients is immense. This evolving approach not only improves recovery outcomes but also enhances the overall quality of life for individuals affected by Long COVID.

Dr. Ke Cheng and colleagues are making significant strides in the development of inhalable exosome therapies for lung and heart repair. In lung treatment, they have pioneered therapies using lung spheroid cell-derived exosomes (LSC-Exo), which have demonstrated remarkable efficacy in treating lung injuries, pulmonary fibrosis, and viral infections such as SARS-CoV-2 [56,57]. LSC-Exo therapy enhances tissue repair, reduces fibrosis, and restores normal lung function, outperforming other cell-based treatments. For cardiac repair, Cheng’s team developed a novel, noninvasive, and repeatable method called stem cell–derived exosome nebulization therapy (SCENT), which delivers exosomes through inhalation following myocardial infarction (MI) [58]. In a mouse MI model, SCENT improved heart function, reduced fibrosis, and promoted cardiomyocyte proliferation by modulating key metabolic pathways, such as glucose metabolism in endothelial cells. This method presents a noninvasive, repeatable treatment for cardiac injury and opens new avenues for clinical application in heart disease. Additionally, Cheng’s research highlights the potential of lung-derived extracellular vesicles (Lung-Exo) as a delivery system for drugs and mRNA therapies, demonstrating superior bioavailability and therapeutic outcomes in both respiratory and cardiovascular conditions [59]. This innovative approach shows great promise for the future of inhalable exosome-based treatments.

Dr. Mengshu Cao introduced the clinical and radiological comprehensive assessment as the primary tool for diagnosing acute exacerbations of interstitial lung disease (AE-ILD) [60]. She also discussed how deep machine learning models applied to clinical and radiological data could aid in the early diagnosis of AE-ILD. In terms of treatment, high-flow nasal cannula (HFNC) and non-invasive positive pressure ventilation (NIPPV) may be effective respiratory management strategies for AE-ILD patients. While corticosteroids remain the mainstay treatment for AE-IPF/ILD, the optimal dosage and duration require further research. Immunosuppressants and biologics may show efficacy for CTD-AE-ILD, though lung transplantation remains the most effective treatment option for AE-ILD patients at present.

## 9. Mechanism and Treatment of Pulmonary Fibrosis

Impaired self-renewal and differentiation of AT2 cells play a significant role in abnormal injury repair and the pathogenesis of IPF. Dr. Jiurong Liang reviewed their seminal finding that AT2 cells are significantly reduced in the lungs of patients with IPF, and the remaining AT2 cells in the IPF lung exhibit impaired renewal capacity [61]. Liang discussed how dysregulation of zinc metabolism may contribute to impaired AT2 cell renewal in IPF [62]. A deficiency in the zinc transporter ZIP8 (encoded by *SLC39A8* gene) in human IPF-derived AT2 cells was found to be associated with reduced AT2 renewal capacity and increased pulmonary fibrosis. ZIP8 is highly and specifically expressed in AT2 cells in healthy lungs. ZIP8-dependent zinc metabolism regulates AT2 progenitor cell renewal via the sirtuin signaling pathway [63,64]. Loss of ZIP8 in IPF AT2 cells resulted in intracellular zinc deficiency and impaired SIRT1 activity, which disrupted AT2 progenitor function. Zinc treatment and SIRT1 activation promoted the self-renewal and differentiation of both human IPF-derived and aged mouse-derived AT2 cells. In contrast, ZIP8 deletion in murine AT2 cells led to impaired renewal, increased susceptibility to spontaneous pulmonary fibrosis, and worsened injury response to bleomycin. These findings suggest that restoring zinc metabolism in AT2 cells may be a promising therapeutic strategy for pulmonary fibrosis.

Despite the unclear etiology of IPF, several genetic factors, including rare variants and single nucleotide polymorphisms (SNPs), have been linked to IPF susceptibility. Among these, Toll-interacting protein (TOLLIP) has emerged as a key player in IPF pathogenesis. TOLLIP is involved in autophagy, inflammation regulation, and intracellular vesicular transport, all of which are relevant to pulmonary diseases. Dr. Yingze Zhang and colleagues identified novel genetic variants in TOLLIP and SPPL2C associated with IPF susceptibility in a GWAS [65,66]. Specifically, the TOLLIP variant rs5743890 was linked to an increased risk of developing IPF and higher mortality rates among patients. Lower TOLLIP expression in IPF lungs, particularly in fibrotic areas, suggests its dual role—protective in early stages and detrimental in later stages of fibrosis [66]. Additionally, SNPs in matrix metalloproteinase 7 (MMP7), a serum marker for IPF, contributed to disease progression. MMP7 levels, influenced by environmental factors such as PM2.5 exposure, exacerbate IPF by reducing DNA methylation and impairing lung function [67]. These findings underscore the complex genetic and environmental interplay in IPF, providing a foundation for future personalized therapeutic strategies.

Dr. Yang Zhou used murine bleomycin models of lung fibrosis, human lung tissues and primary human cells from IPF patients as well as patients with Hermansky-Pudlak Syndrome (HPS), to explore the relationship between CHI3L1 and CRTH2, a receptor expressed on macrophages as well as various type 2 immune cells including ILC2 cells. Zhou’s research demonstrated that CHI3L1 induces monocyte differentiation into a profibrotic macrophage phenotype via the CRTH2 receptor [68]. In patients with HPS types 1 and 4, pulmonary fibrosis develops in the fourth or fifth decade of life and is the major cause of morbidity and mortality. Dr. Zhou’s current research examined the effect of CHI3L1-CRTH2 interactions on ILC2s and the contribution of ILC2-fibroblast crosstalk in the development of pulmonary fibrosis in HPS. These findings demonstrate that ILC2-mediated, CRTH2-dependent mechanism(s) contributes to optimal CHI3L1-induced fibroproliferative repair in HPS-associated pulmonary fibrosis [69]. Dr. Zhou further discussed that phospholipid scramblase-1 (PLSCR1), a type II transmembrane protein, binds to CRTH2 on ILC2s, and the effects of PLSCR1 on ILC2 are mediated through interactions with CRTH2. Therefore, PLSCR1 plays an essential role in the pathogenesis of ILC2 responses, providing new, critical insights into biology and disease pathogenesis and identifying novel targets that can be manipulated in attempts to control lung fibrosis and type 2 immune response in chronic diseases such as asthma [70].

Dr. Ying Xi highlighted the role of the TNFSF-TNFRSF signaling pathway in pulmonary fibrosis, particularly in alveolar repair. In IPF lungs, TNFRSF expression is significantly increased in fibroblasts, but its knockout exacerbates bleomycin-induced pulmonary fibrosis and weakens alveolar epithelial repair. Macrophage-derived TNFSF binds to TNFRSF on fibroblasts, inhibiting their activation while inducing chemokine secretion, which recruits blood monocytes to the lung and promotes their differentiation into alveolar macrophages. These macrophages, particularly in their intermediate differentiation state, facilitate the repair of alveolar epithelial damage. The findings highlight the crucial role of the TNFSF-TNFRSF signaling pathway in alveolar repair, positioning it as a key target for pulmonary fibrosis treatment.

The meeting concluded with a keynote speech by Dr. Dianhua Jiang titled *“The Evolving Paradigms of IPF Pathogenesis: What is Hot, What is Out, and What is in Doubt”*. Dr. Jiang highlighted the evolving paradigms in IPF pathogenesis over the last 30 years, shedding light on current research trends, outdated concepts, and areas that still require further exploration. Once considered promising for IPF patients, extrapulmonary stem cell therapy is no longer viewed as viable, with earlier evidence debunked. While historically regarded as a primary driver of IPF, the roles of inflammation are more complex. The current consensus suggests that the role of inflammation in IPF pathogenesis is highly dependent on cell-cell interactions and the dynamics of cell-niche relationships. The roles and source heterogeneity of AT2 cells, transitional AT2 cells, and basal/basaloid cells are major research focuses on understanding IPF pathogenesis [71,72]. There is growing interest in how aging impacts cellular metabolism and mitochondrial function in AT2 cells and other cell types in IPF [73]. Glycolysis and lipid metabolism have emerged as critical processes in both fibroblasts and AT2 cells in IPF. Despite extensive efforts to explore the diversity and plasticity of fibroblast lineages in IPF, new technologies and innovative approaches continue to advance research into fibroblast subsets, transcriptional regulation, transition states, and activation states. On the contrary, some concepts, such as epithelial-mesenchymal transition (EMT), once considered a significant contributor to the pathogenic fibroblast pool in IPF, are now seen as outdated in IPF pathogenesis.

## 10. Concluding Remarks

The IRM2024 conference showcased recent advancements in respiratory research, fostering a dynamic exchange of ideas among scientists from diverse disciplines. Spanning a broad range of topics, the sessions integrated insights from basic, translational, and clinical research, highlighting recent breakthroughs, addressing ongoing challenges, and exploring future directions for the field.

Despite substantial investments in developing new diagnostic and therapeutic strategies, existing treatments do not always yield favorable outcomes for all patients. However, emerging approaches—such as single-cell multi-omics—are driving biomarker discovery, a crucial step toward the realization of precision medicine. By tailoring treatments to an individual’s genetic, behavioral, and environmental factors, precision medicine shifts away from a one-size-fits-all approach, enabling more personalized care. Although significant progress has been made in precision medicine for conditions like lung cancer and asthma, a substantial unmet need remains for its broader implementation across other respiratory diseases.

The conference emphasized the importance of interdisciplinary and international collaboration, underscoring the need for collective efforts to confront the growing global burden of chronic respiratory diseases. The insights derived from IRM2024 are poised to guide future research and therapeutic strategies, reinforcing the medical community’s commitment to advancing patient care in respiratory health. Through continued collaboration and innovation, we can deepen our understanding and improve the management of these complex conditions, ultimately contributing to better global health outcomes.
